# Deletion of interferon-γ delays onset and severity of dacryoadenitis in CD25KO mice

**DOI:** 10.1186/ar4077

**Published:** 2012-11-01

**Authors:** Flavia SA Pelegrino, Eugene A Volpe, Niral B Gandhi, De-Quan Li, Stephen C Pflugfelder, Cintia S de Paiva

**Affiliations:** 1 Ocular Surface Center, Department of Ophthalmology, Cullen Eye Institute, Baylor College of Medicine, Houston, Texas 77030, USA

## Abstract

**Introduction:**

To investigate the role of interferon-gamma (IFN-γ) in the onset and severity of dacryoadenitis in the CD25 knockout (KO) mouse model of Sjögren Syndrome.

**Methods:**

CD25/IFN-γ double KO (γDKO) mice were created by crossbreeding CD25KO and IFN-γKO mice. Mice were used at 8, 12, and 16 weeks. Lacrimal gland (LG) infiltrating lymphocytes were characterized with flow cytometry. Tear epidermal growth factor (EGF) concentration was measured with enzyme-linked immunosorbent assay (ELISA). Quantitative polymerase chain reaction (PCR) evaluated T-cell-related cytokines in LGs. Serum autoantibodies against M3R in LG lysates were detected with Western blot.

**Results:**

γDKO LG showed lower lymphocytic infiltration at 8 weeks than in the CD25KO parental strain (˜20% versus ˜60%, respectively), which increased to CD25KO levels at 16 weeks. Flow-cytometry analysis showed an increase in CD4^+ ^and CD8^+ ^T cells with aging in γDKO LG, similar to that in CD25KO. γDKO had lower levels of interleukin (IL)-17A, transforming growth-factor (TGF)-β1, IL-21, and CCL20, and higher IL-1β and IL-13 mRNA transcripts in the LG than in the parental CD25KO strain. Autoantibodies to M3R were observed in both strains and significantly increased with aging in both strains. CD25KO mice had very low tear EGF concentrations at all ages, whereas the ear EGF concentration in γDKO mice significantly decreased with aging and inversely correlated with the presence of M3R autoantibodies and the degree of LG CD4 and CD8^+ ^T-cell infiltration.

**Conclusions:**

The deletion of IFN-γ in the CD25KO mice strain delays glandular destruction and preserves glandular function. M3R autoantibodies increased with aging in both the γDKO and the CD25KO strains. The decrease in LG function in γDKO correlated with the degree of T-cell infiltration and the presence of M3R autoantibodies.

## Introduction

Sjögren Syndrome (SS) is a severe autoimmune disease that causes inflammation and dysfunction of the lacrimal and salivary glands. The glandular immunopathology is characterized by multifocal mononuclear cell infiltration, initially around the ductules and later surrounding and replacing secretory acinar cells, leading to decreased secretory function [1,2].

Several mouse strains have been used to study the pathogenic mechanisms of SS. These include the nonobese diabetic (NOD), MRL/Lpr, NZB/W F1 mouse, and transforming growth factor (TGF)-β1 and CD25 knockout (KO) strains [3-11]. The CD25KO mouse develops multiorgan inflammatory disease, inclusive of exocrine glands and gastrointestinal tract, and a profound hemolytic anemia [12,13]. The spontaneous dacryoadenitis that develops in these mice worsens rapidly with age, with a moderate infiltration at age 8 weeks that progresses to complete atrophy and periductal fibrosis at age 16 weeks [14,15].

IFN-γ is secreted by T cells and natural killer cells. This cytokine plays a crucial role in the pathogenesis of a number of immune and inflammatory diseases [16-19]. In SS, it has been shown that IFN-γ is an early regulator of the acinar cell pathology by inhibiting the G_1 _phase of the acinar cell cycle, altering integrin expression and decreasing cell viability [5]. It has also been implicated in the cornification of the conjunctival epithelia in a murine mouse model of dry eye [20]. Several lines of evidence indicate that the T-helper (Th)-1 cytokine IFN-γ is associated with the pathogenesis of SS. High concentrations of serum IFN-γ and high levels of IFN-γ mRNA in the conjunctiva were found in SS patients [21,22]. Expression of high levels of IFN-γ mRNA in labial salivary gland biopsies from SS patients was found to correlate with the degree of T-cell infiltration [23].

Alternative explanations for exocrine dysfunction include epithelial cell apoptosis, direct effects of cytokines, autoantibodies against nuclear proteins (ANAs), ribonuclear proteins (Ro/SSA and La/SSB), α-fodrin, and the muscarinic acetylcholine type 3 receptor (M3R) or dysregulation of the parasympathetic nervous system [24]. Previous studies have shown that repeated injection of sera from SS patients into mice caused a 40% to 60% decrease in salivary secretory function [25].

The purpose of this study was to investigate the effects of IFN-γ deletion on the pathogenesis of the dacryoadenitis that develops in CD25KO mice.

## Materials and methods

### Animals

This research protocol was approved by the Baylor College of Medicine Center for Comparative Medicine, and it conformed to the standards in the ARVO Statement for Use of Animals in Ophthalmic and Vision Research.

Heterozygous breeder pairs of CD25KO (B6.129S4-*Il2ra^tm1Dw^*/J) and IFN-γKO (B6.129S7-*Ifng^tm1Ts^*/J) mice in a C57BL/6 background were purchased from Jackson Laboratories (Bar Harbor, ME, USA) for establishing breeder colonies.

To create a CD25/IFN-γ double KO (γDKO), IFN-γKO mice were mated with heterozygous CD25 mice. F_1 _was genotyped, and double-heterozygous CD25^+/- ^and IFN-γ^+/- ^mice were mated. F_2 _offspring were genotyped, and the mice that were CD25^+/- ^and IFN-γ^-/- ^were used as breeder pairs to generate CD25/IFN-γ double-KO (γDKO) mice.

The genotype of gene-knockout strains was confirmed by using a previously reported protocol [14]. Mice were used at ages 8, 12, and 16 weeks. A minimum of 23 animals per time point (8, 12, and 16 weeks) per strain (CD25KO, IFN-γKO, γDKO) were used: five for histologic sections, 10 for flow-cytometry analysis, and eight for gene-expression studies. In some strains/time point, up to 20 animals were used for flow cytometry.

Neither the CD25KO parental strain nor the IFN-γKO strain has a gender bias in their pathology [9]. In our preliminary evaluation, we did not observe a gender difference in severity of LG infiltration in the newly created γDKO strain. Therefore, the data represent an average of both genders (1:1) for each parameter/age.

### Histology, periodic acid-Schiff staining, and immunohistochemistry

Extraorbital lacrimal glands, submandibular glands, small intestine, kidneys, and eyes were excised, fixed in 10% formalin, and paraffin embedded, and 8-μm sections were cut. Sections were stained with hematoxylin and eosin (H&E) for evaluating morphology. The area of lymphocytic infiltration was circumscribed in digital images of H&E-stained sections, as previously described [9]. In brief, digital images were analyzed by using NIS Elements Software: areas of lymphocytic infiltration were circumscribed, as well as the total area of the lacrimal gland. The percentage infiltration was calculated as area of infiltration × 100/total area.

For immunohistochemistry, extraorbital LGs from each strain/time point (*n *= 5) were excised, embedded in optimal cutting-temperature compound (VWR, Suwanee, GA, USA), and flash frozen in liquid nitrogen. Sagittal 8-μm sections were cut with a cryostat (HM 500; Micron, Waldorf, Germany) and placed on glass slides that were stored at -80°C.

Immunohistochemistry in LG cryosections was performed by using CD4 (BD Pharmigen, San Jose,CA, clone H129.9, 10 μg/ml), CD8α (BD Pharmigen,San Jose,CA clone 53e6.7, 3.125 μg/ml), or CD19 (Abcam, Cambridge, MA clone 6D5, 2 μg/ml) antibodies. Staining was performed with appropriate biotinylated secondary antibodies (all from BD Pharmingen) and a Vectastain Elite ABC kit with Nova Red reagents (Vector, Burlingame, CA, USA). Secondary antibody alone and appropriate anti-mouse isotype (BD Bioscience, San Diego, CA, USA) were used as controls. Six sections from each animal/group/time point were examined and photographed.

### Flow-cytometry analysis of infiltrating cells

Single-cell suspensions of LGs from C57BL/6, CD25KO, IFN-γKO, and γDKO mice at ages 8, 12, and 16 weeks (minimum of eight up to 20/strain/age) were prepared as previously reported [9]. In brief, extraorbital lacrimal glands were excised, minced, and subjected to collagenase digestion for 1 hour at 37°C under constant agitation. Collagenase was neutralized by adding complete RPMI with FBS; cells were filtered by using a 70-μm cell strainer, centrifuged, and resuspended. Cells were stained with anti-CD16/32 (BD Pharmingen), followed by cell-surface staining with FITC-conjugated anti-CD4 (GK 1.5), PE-anti-CD8 (clone 53-6.7), or APC-B220 (clone RA3-6B2). Negative controls were stained with isotype antibodies (BD Pharmingen). Live/dead cell exclusion was visualized with propidium iodide. A BD LSRII Bench-top cytometer was used for cytometry, and data were analyzed by using FlowJo Software (Tree Star Inc.).

### RNA isolation and quantitative PCR

Extraorbital LGs from CD25KO, IFN-γKO, and γDKO were excised, and total RNA was extracted by using the Arcturus PicoPure RNA Isolation Kit (Applied Biosystems, Foster City, CA, USA), quantified by a NanoDrop ND-1000 Spectrophotometer (Thermo Scientific, Wilmington, DE, USA), and stored at -80°C. Eight samples per strain/age were used, and one sample consisted of pooled glands from the same animal. Samples were treated with DNase to eliminate genomic DNA contamination (Qiagen, Valencia, CA, USA). First-strand cDNA was synthesized with random hexamers by M-MuLV reverse transcription (Ready-To-Go You-Prime First-Strand Beads; GE Healthcare, Inc., Arlington Heights, NJ, USA), as previously described [9].

Quantitative real-time PCR was performed with specific MGB probes (Taqman; Applied Biosystems) and PCR master mix (Taqman Gene Expression Master Mix), in a commercial thermocycling system (StepOnePlus Real-Time PCR System; Applied Biosystems). Murine MGB probes were IL-17A (Mm00439619), IFN-γ (Mm00801778), IL-13 (Mm00434165), IL-21 (Mm00517640), CCL20 (Mm00444228), TGF-β1 (Mm00441724), GATA-3 (Mm00484683), RORγT (Mm00441139), and IL-1β (Mm00434228).

The copy number of the gene of interest was calculated by comparing the sample with the gene-specific standard curve, previously prepared by using commercial mouse cDNA (Zyagen, San Diego, CA, USA). Samples and standards were assayed in duplicate. A nontemplate control and total RNA without retrotranscription were included in all the experiments to evaluate PCR and DNA contamination of the reagents used.

### Tear washings and EGF enzyme-linked immunosorbent assay

Tear-fluid washings were collected from eight animals/strain/age and from eight young C57BL/6 mice, by using a previously reported method [9]. One sample consisted of tear washings from both eyes of one mouse pooled (2 μl) in PBS+0.1% BSA (8 μl) and stored at -80°C until the assay was performed.

The EGF concentration in tear samples was assayed with a commercial ELISA kit according to the manufacturer's protocol (R&D Systems, Minneapolis, MN, USA). The microplate was read by using an ELISA-reader instrument (Tecan Infinite M200) equipped with Magellan V6.55 software. Biologic replicate samples were averaged. Results are presented as mean ± standard deviation (pictograms per milliliter).

### SSA and SSB autoantibodies ELISA

Serum was collected by cardiac puncture after death from eight animals/strain/age and from eight young C57BL/6 mice. SSA and SSB were individually assayed in serum by using commercially available ELISA kits (Calbiotech, Spring Valley, CA, USA) according to the manufacturer's instructions.

### Western blot

C57BL/6 extraorbital LGs (*n *= 4 animals) were excised and lysed in RIPA buffer containing a protease inhibitor cocktail tablet. Total protein concentrations were measured with a Micro BCA protein kit (Pierce, Rockford, IL, USA), and 200 μg of protein/sample was separated with SDS-polyacrylamide gel electrophoresis, blocked, and incubated overnight at 4°C with either a commercial antibody raised against M3R (4 μg; Santa Cruz Biotechnology) or serum from CD25KO, γDKO, and C57BL/6 mice (diluted at 1:1,500), and appropriate HRP-conjugated secondary antibodies were applied (Pierce; 1:2,000). Signals were detected by using the ECL Plus Detection Reagents (Amersham Place, Little Chalfont, Buckinghamshire, UK), and the images were acquired and analyzed with a Kodak Image Station 2000R (Eastman-Kodak, New Haven, CT, USA). Specificity of the antibody was confirmed with preincubation assays with the same peptide used to generate the antibody. Loading control studies were performed by immunoblotting all four LG lysates with a commercial antibody anti-β actin (Sigma-Aldrich; 1:5,000 dilution). Location of an immunodetected band in LG lysates was compared with a positive control (brain lysates; Santa Cruz Biotechnology). Net bands intensities were measured with Kodak 1D v3.6 software in digital images.

### Co-immunoprecipitation procedure

Four and eight micrograms of commercial affinity-purified goat polyclonal antibody anti-M3R or 20 and 40 μl each of two sera from two 16-week-old CD25KO mice were covalently cross-linked to 50 μl of Dynabeads protein G (Invitrogen, Dynal AS, Oslo, Norway) according to the manufacturer's protocol. Proteins from two different mouse whole-LG cell lysates (100 to 200 μg) were used as an antigen-containing sample. The immunoprecipitated Dynabeads complexes were washed and separated on a Dyna-Mag-2 magnet. Eluted proteins and washes were processed for Western blot analysis. When samples were immunoprecipitated by using the M3R antibody, they were further blotted by using the CD25KO serum and vice versa.

### Statistical analysis

The Kruskal-Wallis test was used to determine overall statistical significance followed by a two-tailed *t *test for individual differences in gene expression, EGF concentration, and CD4/CD8 infiltration. Nonlinear regressions were used to evaluate the correlation of tear EGF concentration versus muscarinic 3-receptor densities and CD4^+ ^T-cell and CD8^+ ^T-cell percentages by using data collected from all the strains. Statistical significance was calculated by Spearman correlation, which makes no assumption about the normality of data. *P *≤ 0.05 was considered statistically significant. These tests were performed by using GraphPad Prism 5.0 software (GraphPad Software Inc., San Diego, CA, USA).

## Results

### Deletion of IFN-γ delays the onset of dacryoadenitis in CD25KO mice

Previous studies in the NOD spontaneous SS model showed that IFN-γ has a critical role in the submandibular but not in lacrimal-gland disease [5]. The offspring of γDKO mice appeared normal at birth and after weaning. Similar to parental CD25KO mice, enlargement of lymphoid tissues were observed macroscopically, and microscopic lymphocytic infiltration of small intestine, submandibular glands, and kidneys was observed in DKO mice, although to a lesser extent than in the CD25KO parental strain (data not shown).

**Figure 1:**
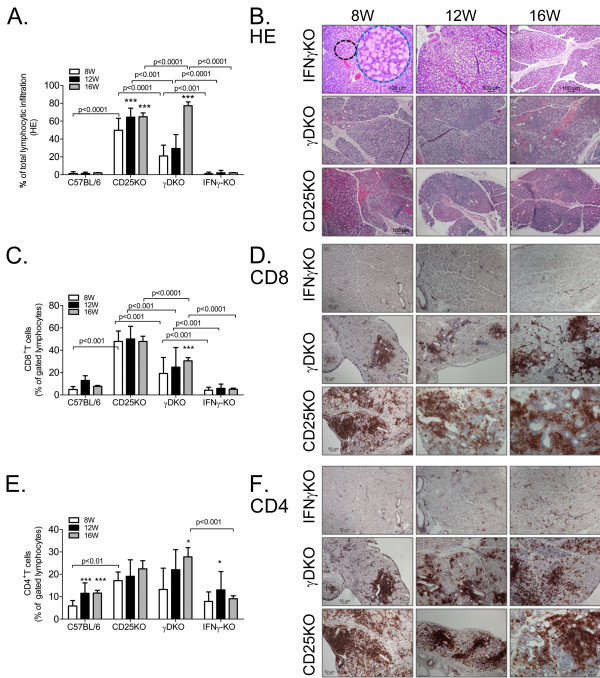
**Histology and T-cell infiltration in lacrimal gland (LG)**. **(A) **Total cell infiltration measured in digital images of hematoxylin/eosin (H&E)-stained LG sections of C57BL/6, CD25KO, γDKO, and IFN-γKO mice at ages 8, 12, and 16 weeks (8W,12W,16W, respectively). **(B) **Representative images of H&E-stained LGs from mice aged 8 to 16 weeks. The blue-dotted circle is a higher magnification of the area demarcated by the black dotted circle in IFN-γKO mice at age 8 weeks. Scale bar, 100 μm. **(C) **Bar graphs showing mean ± standard deviation of percentage of CD8^+ ^T cells gated in LG isolates with flow cytometry in C57BL/6, CD25KO, γDKO, and IFN-γKO mice at ages 8, 12, and 16 weeks. Data are presented as mean ± standard deviation of 10 to 20 individual samples/strain/time point. **(D) **Representative images of immunohistochemistry for CD8-positive T cells in the LGs of all three strains at different ages. Positive cells are stained red. Scale bar, 50 μm. **(E) **Bar graphs showing mean ± standard deviation of percentage of CD4^+ ^T cells gated in LG isolates with flow cytometry in C57BL/6, CD25KO, γDKO, and IFN-γKO mice at ages 8, 12, and 16 weeks. Data are presented as mean ± standard deviation of 10 to 20 individual samples/strain/time point. **(F) **Representative images of immunohistochemistry for CD4-positive T cells in the LGs of IFN-γKO, γDKO, and CD25KO mice at ages 8, 12, and 16 weeks. Positive cells are stained red. Scale bar, 100 μm. *P < 0.05; **P < 0.01; ***P < 0.001 intrastrain comparison versus age 8 weeks.

Macroscopically, we observed the γDKO LGs to have normal appearance and size at 8 weeks of age, similar to the IFN-γKO and C57BL/6. Young CD25KO LGs showed acinar atrophy, periductal fibrosis, and intense lymphocytic infiltration occupying up to 50% of the total area of the gland (Figure 1A, B). The dacryoadenitis in CD25KO worsened with age, progressing to total acinar loss, ductal proliferation, and atrophy at age 16 weeks. Compared with the CD25KO parental strain, young γDKO mice showed less cell infiltration (˜20%), with preservation of architecture and normal-appearing acini occupying about 70% of LGs, with progressive increase in the area of lymphocytic infiltration in the LGs with aging, reaching 80% of total LG area at 16 weeks, whereas the remainder of the LG was atrophic with few normal acini remaining (Figure 1A, B). Comparatively speaking, the appearance of γDKO 16W LG resembled CD25KO 12W LG regarding the amount of inflammation that was seen. IFN-γKO LGs had minimal lymphocytic infiltration at all time points.

### Analysis of the lymphocytic populations infiltrating the lacrimal glands of CD25KO and γDKO

The better to characterize the lymphocytic infiltration, LG sections from all groups were immunostained for CD4^+^, CD8^+ ^T cells, and B cells and quantified with flow cytometry (Figure 1 and see Additional file 1).

Flow cytometry showed a predominance of CD8^+ ^T cells over CD4^+ ^T cells in both γDKO and CD25KO LG at age 8 weeks of age (Figure 1C and Additional file 1). We observed that γDKO-8W showed less T-cell infiltration than the CD25KO and IFN-γKO parental strains, but showed a progressive increase in CD4^+ ^and CD8^+ ^cells (Figure 1C, E), in roughly equal proportions with aging, mainly around the epithelial ducts (Figure 1D, F). Minimal T-cell infiltration was observed in control C57BL/6 ([15] and data not shown) and IFN-γKO LG (Figure 1D, F). CD4^+ ^T-cell infiltration was observed in the same areas as CD8 infiltration in all strains (Figure 1F) and was noted to increase with age in both CD25KO and γDKO strains (Figure 1E). Flow cytometry (Additional file 1) confirmed a progressive increase of CD4^+ ^T cells with aging in these strains, reaching statistical significance in γDKO at age 16 weeks (Figure 1E). The increase in CD4^+ ^T cells in γDKO was accompanied by an increase in CD8^+ ^T cells (Figure 1C, E).

**Figure 2:**
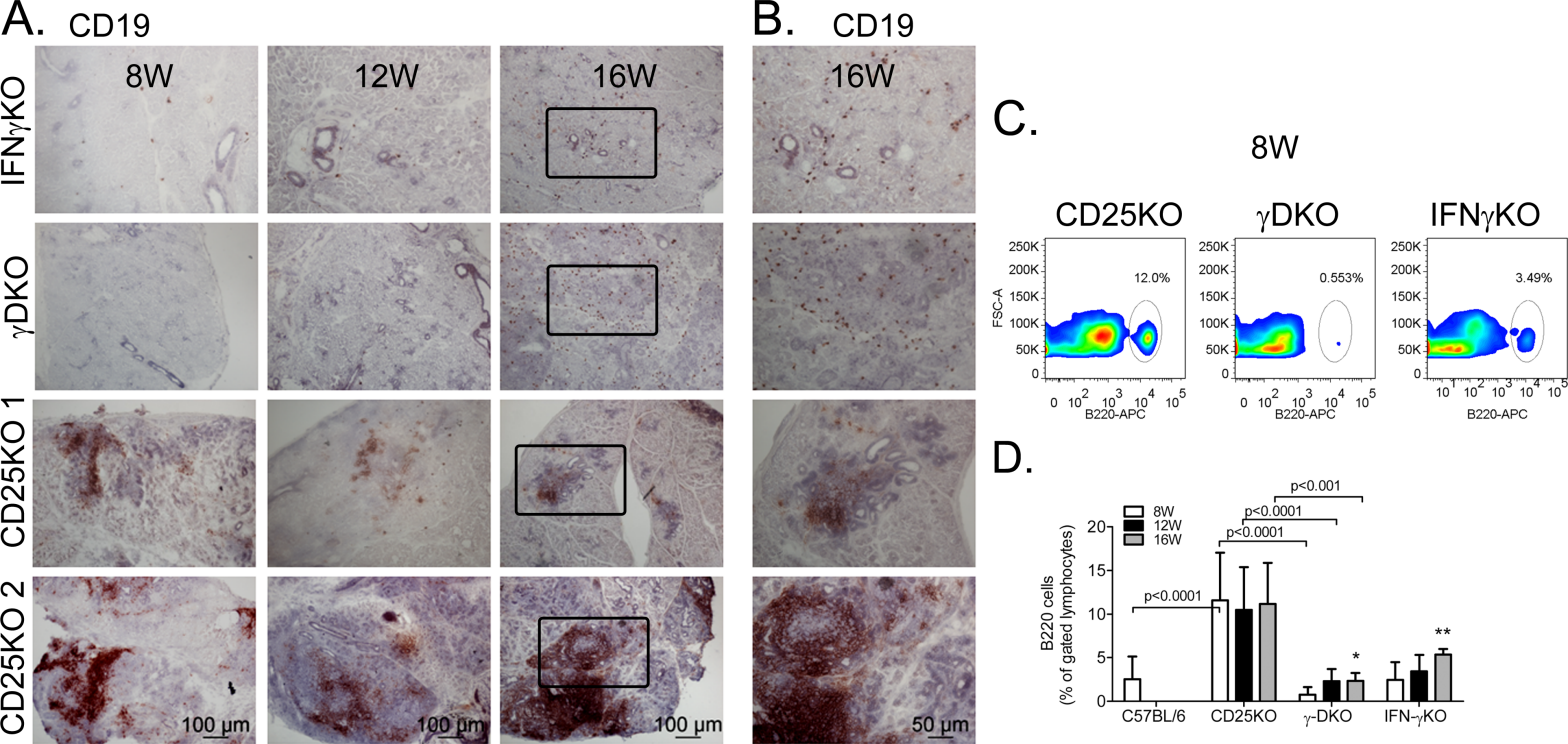
**B-cell infiltration in lacrimal gland (LG)**. **(A) **Representative images of CD19^+ ^cells in the LGs of IFN-γKO, γDKO, and CD25KO mice at ages 8, 12, and 16 weeks (8W,12W,16W, respectively). CD25KO 1 and 2 are two different animals of the same age. Positive cells are stained red. Scale bar, 100 μm. **(B) **High magnification of black square in LG at age 16 weeks in all three strains. Scale bar, 50 μm. **(C) **Representative flow-cytometry plots of freshly isolated cells from LGs of different strains stained with B220-APC conjugated antibody **(x axis) **versus forward-scatter area **(**FSC-A**, y axis)**. Lymphocytes were gated based on characteristic light-scatter properties; single lymphocytes were gated based on forward-scatter height versus forward-scatter area (FSC-A), and dead cells were excluded with propidium iodide. **(D) **Bar graph showing mean ± standard deviation of percentage of B220^+ ^cells gated in LG isolates with flow cytometry at ages 8, 12, and 16 weeks (8W,12W,16W, respectively). *P < 0.05 intrastrain comparison; **P < 0.01 intrastrain comparison versus age 8 weeks.

Immunohistochemistry for B cells by using the CD19 antibody showed that few sparse infiltrating CD19^+ ^cells were seen in IFN-γKO and γDKO LG at ages 8 and 12 weeks (Figure 2A); whereas they were slightly more frequent at age 16 weeks (Figure 2). In the CD25KO strain, the presence of B cells was much more variable within the same age group; two types of infiltration patterns were observed. The first pattern resembled the one observed in the IFN-γKO and γDKO LGs, whereas B cells were dispersed. The second was suggestive of germinal center formation within the LG (Figures 2A and 2B), and it was seen only in the CD25KO mouse strain. Both patterns were seen with similar frequency in the CD25KO LGs.

Flow-cytometry analysis (Figure 2C) confirmed the increase in B220^+ ^cells at age 16 weeks in both γDKO and IFN-γKO, and the increased amount of infiltrating B cells in CD25KO at all ages when compared with γDKO (Figure 2D).

### Cytokine profile in CD25KO, IFN-γKO, and γDKO

**Figure 3:**
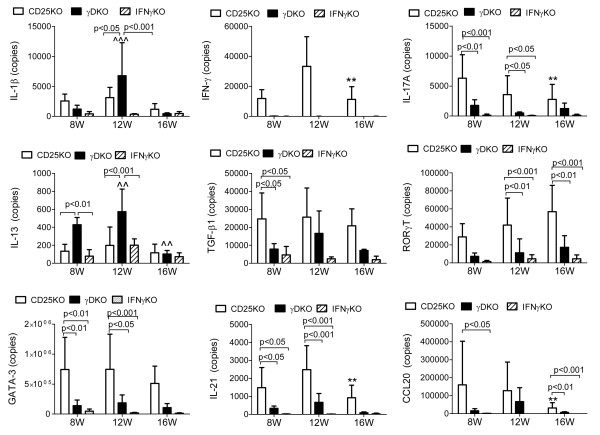
**Quantitative gene analysis of mRNA copies in lacrimal gland**. T-helper-associated cytokines and transcription factors and proinflammatory cytokine IL-1β were investigated in lacrimal glands of CD25KO, γDKO, and IFN-γKO mice at ages 8, 12, and 16 weeks. Bars indicate mean ± standard deviation of number of copies from eight individual samples. **P < 0.01 intrastrain CD25KO comparison; ^^ and ^^^, *P *< 0.01 and *P *< 0.001 intrastrain γDKO comparison versus age 8 weeks, respectively.

Previous studies showed that administration of IL-1 to LGs induces release of proinflammatory cytokines, induces lymphocytic infiltration, and decreases LG function [26,27]. Real-time PCR showed that *IL-1β *mRNA transcripts were present at low levels at age 8 weeks in all three strains, CD25KO, γDKO, and IFN-γKO. At age 12 weeks, a significant increase was noted in the γDKO strain (Figure 3). It is interesting to note that the peak of *IL-1β *accompanied the increase in lymphocytic infiltration in the LGs of γDKO mice, a finding that we also observed in our previously reported study showing progressive CD4^+ ^T-cell infiltration of the LGs after pharmacologic blockade of LG secretion [28].

To characterize the T-helper phenotype associated with dacryoadenitis, we evaluated levels of mRNA transcripts encoding Th-1, Th-2, and Th-17-associated genes by using quantitative real-time PCR in CD25KO and γDKO mice. As a quality control, *IFN-γ *was assayed in LGs of γDKO and IFN-γKO mice. As previously published [9], *IFN-γ *mRNA levels were elevated in the parental CD25KO strain compared with C57BL/6 control mice (data not shown), but they were absent in γDKO and IFN-γKO (Figure 3). A significant increase in the level of the transcripts of the IFN-γ was observed in CD25KO at 12W, with a subsequent decrease at 16W.

Significantly higher levels of Th-17-pathway genes (*IL-17A, TGF-β1, IL-21, RORγT, and CCL20*) were found in 8-week-old CD25KO compared with young γDKO mice (Figure 3). Levels of *IL-17A, IL-21*, and *CCL20 *mRNA decreased with time in CD25KO mice, whereas they remained at similar levels in γDKO and IFN-γKO mice. No change was found in TGF-β1 expression with aging.

The level of *IL-13 *mRNA transcript was higher in the γDKO strain at 8 and 12 weeks compared with CD25KO mice, but showed a significant decrease at age 16 weeks. Similar to *IL-1β*, the increase of *IL-13 *mRNA at age 12 weeks paralleled the increase in cell infiltration in LG sections.

### Aging increases muscarinic receptor autoantibodies in CD25KO and γDKO mice

To investigate the presence of autoantibodies, we performed ELISA for SSA and SSB in serum of CD25KO, IFN-γKO, and γDKO mice by using commercially available ELISA kits. No SSA and SSB antibodies were detected in serum from any strain at any age by using these kits (data not shown).

**Figure 4:**
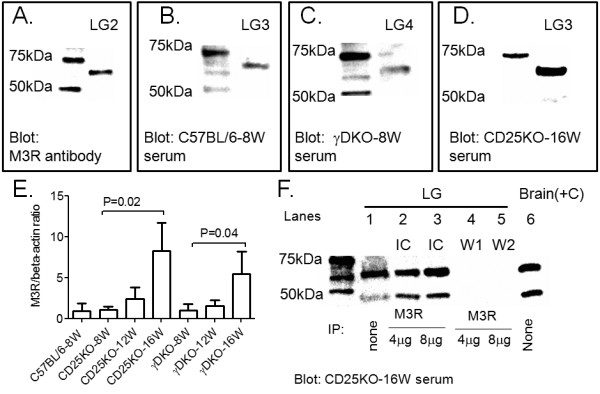
**Western blot analysis of muscarinic 3 receptor**. Extraorbital lacrimal glands (LGs; *n *= 4) were lysed and processed by using different antibodies/serum at all ages. Representative images are shown; the number after "LG" indicates the ID of LG used for that particular Western blot. **(A) **Representative Western blot of LG2 lysates blotted with M3R antibody. **(B through D) **Representative Western blot of LG lysates blotted with C57BL/6 (B, LG3), γDKO (C, LG4), or CD25KO (D, LG3) sera used to generate the bar graph in E of net band densities (arbitrary values). 8W, 8 weeks; 16W, 16 weeks. **(E) **Mean ± standard deviation M3R net band densities normalized by β-actin band densities in Western blots of LG lysates blotted C57BL/6, γDKO, or CD25KO sera at different ages. **(F) **Co-immunoprecipitation studies. LGs were co-immunoprecipitated with either 4 or 8 μg of muscarinic 3 receptor antibody (lanes 2 and 3) and blotted with CD25KO-16W serum. Excess proteins that were not precipitated (Wash 1 (W1) and wash 2 (W2), lanes 4 and 5, respectively) were included in the gel and blotted as negative controls. LGs and brain lysates (lane 1 and 6) that were not immunoprecipitated were included in the gel as positive controls.

Serum autoantibodies to the muscarinic 3 receptor have been detected in a high percentage of patients with SS [29,30] and have been proposed as a marker of dry eye in SS [29]. Because we still observed progression of disease in the absence of IFN-γ, we hypothesized that M3R autoantibodies could be involved in the progressive LG immunopathology with aging. Immunoblotting studies (Figure 4), by using four different C57BL/6 LG cell lysates as the antigen source, identified a major band ˜60 kDa detected with the serum from C57BL/6, CD25KO, and γDKO at age 8 weeks at similar intensities. Loading control with β-actin antibody was also performed (data not shown) and was used to normalize M3R expression. A smaller band ˜45 kDa was occasionally seen (Figure 4F). Aging significantly increased the M3R/β-actin ratio in both CD25KO and γDKO mice (Figure 4E).

Confirmatory immunoprecipitation assays (by using the commercial antibody to pull down M3R antigen (4 μg and 8 μg) from total LG protein lysates and blotted by using the CD25KO serum) showed that M3R antigen that belonged to immune complexes was correctly identified by CD25KO serum, whereas remaining washes containing protein without precipitation yielded no band (washes, lanes 4 and 5 of Figure 4F). These results were confirmed by using the CD25KO serum in the first step of the co-precipitation, and bands were correctly identified in the immunoprecipitate by using the commercial antibody (data not shown).

### Deterioration of LG function with aging

**Figure 5:**
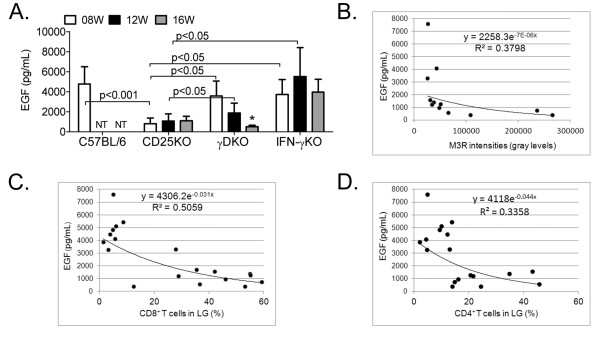
**Lacrimal gland function**. **(A) **Epidermal growth factor (EGF) concentration measured in tear washings from different strains. NT, not tested. **(B through D) **Spearman correlations between tear EGF concentrations and muscarinic 3-receptor band densities (B, measured in Western blot; *P *= 0.001), lacrimal gland (LG) CD8^+ ^T (C, *P *= 0.002), and CD4^+ ^T cells (D, *P *= 0.004). Percentage of CD8 and CD4 infiltration was measured with flow cytometry. R^2^, coefficient of determination.

Because EGF in tears is generally accepted to be exclusively secreted from the LGs, we measured EGF in tears of all mouse strains as a measure of LG function (Figure 5A). We observed that CD25KO mice of all ages had significantly lower EGF concentration in tear washings compared with young C57BL/6 mice, indicating their profound impairment in LG secretion at all time points. Young γDKO mice already showed a significant decrease in EGF concentration, whereas mild to moderate LG infiltration was noted (Figure 1); however, a significantly progressive decrease in levels of tear EGF was observed with aging from 8 to 16 weeks (Figure 5A).

Because the decrease in tear EGF concentration was accompanied by worsening dacryoadenitis and LG destruction, we correlated EGF levels with the factors that also increased with aging (that is, M3R autoantibodies and T-cell infiltration). We observed that tear EGF concentrations showed a significant inverse correlation with M3R band densities, percentage of CD4^+ ^and CD8^+ ^T cells measured with flow cytometry (Figure 5B to D; *P *= 0.001; *P *= 0.004; *P *= 0.002, respectively).

## Discussion

We describe in this study that deletion of IFN-γ delays the onset and severity of dacryoadenitis in the CD25KO mouse strain that develops autoimmune manifestations resembling SS. The autoimmunity in the LGs of the γDKO mouse was accompanied by an increase in CD4^+^, CD8^+ ^T- and B-cell infiltration with aging from ages 8 to 16 weeks, an increase in M3R autoantibodies, and a concomitant decrease in EGF concentration in tears. The infiltration with CD4^+ ^and CD8^+ ^T cells and M3R autoantibodies correlated with the impairment in LG secretory activity by using tear EGF concentration as a measure of function. Lysates of γDKO LG used for gene expression showed a different T-cell-related cytokine profile than the CD25KO parental strain, where a significant peak in *IL-1β *and *IL-13 *expression was observed at age 12 weeks.

Genetic deletion of IFN-γ in CD25KO mice did not abolish development of autoimmunity; rather, it delayed its onset and attenuated the severity. Our results agree with the findings of Peck and colleagues [5] that showed that deletion of IFN-γ and IFN-γ-receptor genes in the susceptible NOD mouse strain reversed the lymphocytic infiltration of the submandibular glands and the salivary secretory dysfunction, but had no effect on the severity of lacrimal gland disease [5]. However, findings in the NOD/IFN-γKO combined with our findings in the γDKO mice suggest that IFN-γ is not entirely responsible for the LG immunopathology in SS.

In this present study, the presence of inflammatory cells correlated with the loss of LG function, by using tear EGF concentration as a measure of function. LGs in young CD25KO mice showed extensive infiltration and EGF levels that were already markedly decreased, whereas the less-severe γDKO mice that had significantly smaller areas of LG infiltration still had measurable tear EGF at 8 weeks, albeit significantly lower than that in C57BL/6 wild-type mice controls. With aging, tear EGF concentration in γDKO mice showed a progressive decrease that paralleled the increase in T-cell infiltration. EGF expression and secretion is regulated differently from fluid production, so it may be appropriate to state that tear EGF is one conveniently obtained measure of LG function. Lacrimal insufficiency is responsible for some severe forms of dry eye and may be caused in part by cytokines such as IL-1 [26,27] that are released by infiltrating cells or stressed glandular epithelial cells [31,32]. Previous studies found a correlation between EGF and the severity of ocular-surface disease [33].

The proinflammatory and apoptotic effects of IL-1 in the LG have been extensively studied by Zoukhri and colleagues [26,34-37]. In one of their experiments, they showed that a single dose of IL-1 into the extraorbital LG induced a mild decrease in LG secretion while inducing a robust yet reversible (7 to 13 days), inflammatory response that led to destruction of lacrimal gland acinar epithelial cells [34]. In our studies, the peak of *IL-1β *(age 12 weeks) preceded the greatest increase in lymphocytic infiltration (age 16 weeks), suggesting that similar to Zoukhri's studies [26,34-37], IL-1β may be critical in inducing an inflammatory response that happens after the peak of IL-1β. Worth noting, all inflammatory cytokines were decreased at age 16 weeks in γDKO LGs, suggesting an atrophic, end stage.

Interestingly, the γDKO mice showed elevated copies of *IL-13 *mRNA at ages 8 and 12 weeks, compared with the CD25KO parental strain. *In vitro *interplay between IFN-γ and IL-13 in the generation of polarized Th-1 and Th-2 CD4^+ ^T cells was previously reported [38]. *In vivo*, the addition of IFN-γ was noted to antagonize the goblet cell hyperplasia induced by IL-13 in lungs [38]. Adoptive transfer of IFN-γ-producing Th-1 cells also suppressed goblet cell hyperplasia in an asthma mouse model [39]. In our experimental dry-eye model, in which wild-type mice are subjected to a drafty low-humidity environment, and lacrimal gland secretion is pharmacologically inhibited [22,40], we demonstrated that IL-13 is a homeostatic factor for conjunctival goblet cells, whereas IFN-γ suppresses maturation of these cells [41]. Adoptive transfer of CD4^+ ^T cells from donor mice subjected to desiccating stress increased corneal epithelial apoptosis; however, topical neutralization of IFN-γ in immunodeficient adoptive transfer recipients prevented corneal epithelial apoptosis [42]. In another experiment, we showed that increased activated-caspase-3 and -8 and TUNEL immunoreactivity were noted in conjunctival epithelia in B6 mice compared with IFN-γKO mice after desiccating stress, and exogenous IFN-γ administration further increased these parameters [43].

We found that both γDKO and CD25KO strains developed increased levels of serum M3R autoantibodies with aging that inversely correlated with tear EGF concentration. The target autoantigen(s) in the dacryoadenitis that develops in the CD25KO strain has not been identified. We did not detect SSA or SSB autoantibodies in the sera of these autoimmune strains, but we did find serum reactivity to LG proteins with an identical size as the M3R, suggesting that this may be a target antigen. Antibodies against M3R were more commonly detected in the serum of patients with primary and secondary SS than in those with other autoimmune diseases, such as rheumatoid arthritis and systemic lupus erythematosus, or healthy subjects. Elegant experiments have shown that administration of human sera to mice causes either a stimulatory or inhibitory effect in lacrimal and submandibular gland secretion [25,29,44,45]. Iizuka and colleagues [46] demonstrated that RAG1^-/- ^adoptive transfer recipients of CD3^+ ^T cells isolated from M3R^-/- ^mice immunized with murine M3R peptides developed LG infiltration and destruction of salivary glands [46].

One major problem in the CD25KO parental strain is the lack of T-regs. Recent studies showed that deficient T-reg cell function may contribute to the development of multiorgan systemic autoimmune disease [47]. Spontaneous development of dacryoadenitis and keratoconjunctivitis is observed in mouse strains with defective T-reg function, such as the CD25KO strain used in these experiments, as well as the TGF-β1KO. In NOD mice, the deletion of T-reg cells worsens autoimmune activity [48].

## Conclusions

Our results show that the deletion of IFN-γ in the CD25KO mouse strain delays glandular destruction and preserves lacrimal glandular function. We also showed that M3R autoantibodies increased with aging in both γDKO and CD25KO strains. Taken together, our findings show that IFN-γ is not the sole contributor in the dacryoadenitis that develops in this SS mouse model and demonstrated, for the first time, the presence of M3R antibodies in the CD25KO mouse strain.

## Abbreviations

ANA: anti-nuclear antibody (anti-Ro/SSA; anti-La/SSB); CD25KO: CD25 knockout; DKO: double knockout; γDKO: CD25/IFN-γ double knockout; EGF: epidermal growth factor; KO: knockout; LG: lacrimal gland; M3R: muscarinic 3 receptor; NOD: nonobese diabetic; SS: Sjögren syndrome; TGF-β1: transforming growth-factor β1; Th: T helper.

## Competing interests

The authors declare that they have no competing interests.

## Authors' contributions

All authors contributed to the final manuscript. FSAP, SCP, DQL, and CSDP participated in the design of the study, statistical analysis and interpretation of the data, drafting the article, and critical revision of the article for important intellectual content. EAV and NBG assisted with collection and acquisition of the data and critically revised the manuscript. All authors read and approved the manuscript for publication.

## Supplementary Material

Additional file 1 **CD4 and CD8 representative flow cytometry in lacrimal glands (LGs)**. Representative flow-cytometry plots of freshly isolated cells from LGs of CD25KO, γDKO, and IFN-γKO mice stained with either CD4-FITC-conjugated antibody **(x axis) **or CD8-PE-conjugated antibody **(y axis) **at ages 8, 12, and 16 weeks (8W,12W,16W, respectively). Lymphocytes were gated based on characteristic light-scatter properties; single lymphocytes were gated based on forward-scatter height versus forward-scatter area (FSC-A), and dead cells were excluded with propidium iodide staining. Click here for file
