# Supplementary figures and images for: Deletion of interferon-γ delays onset and severity of dacryoadenitis in CD25KO mice
Source: Arthritis Res Ther. 2012 Nov 1;14(6):R234. doi: 10.1186/ar4077 (PMC3674599; doi:10.1186/ar4077)

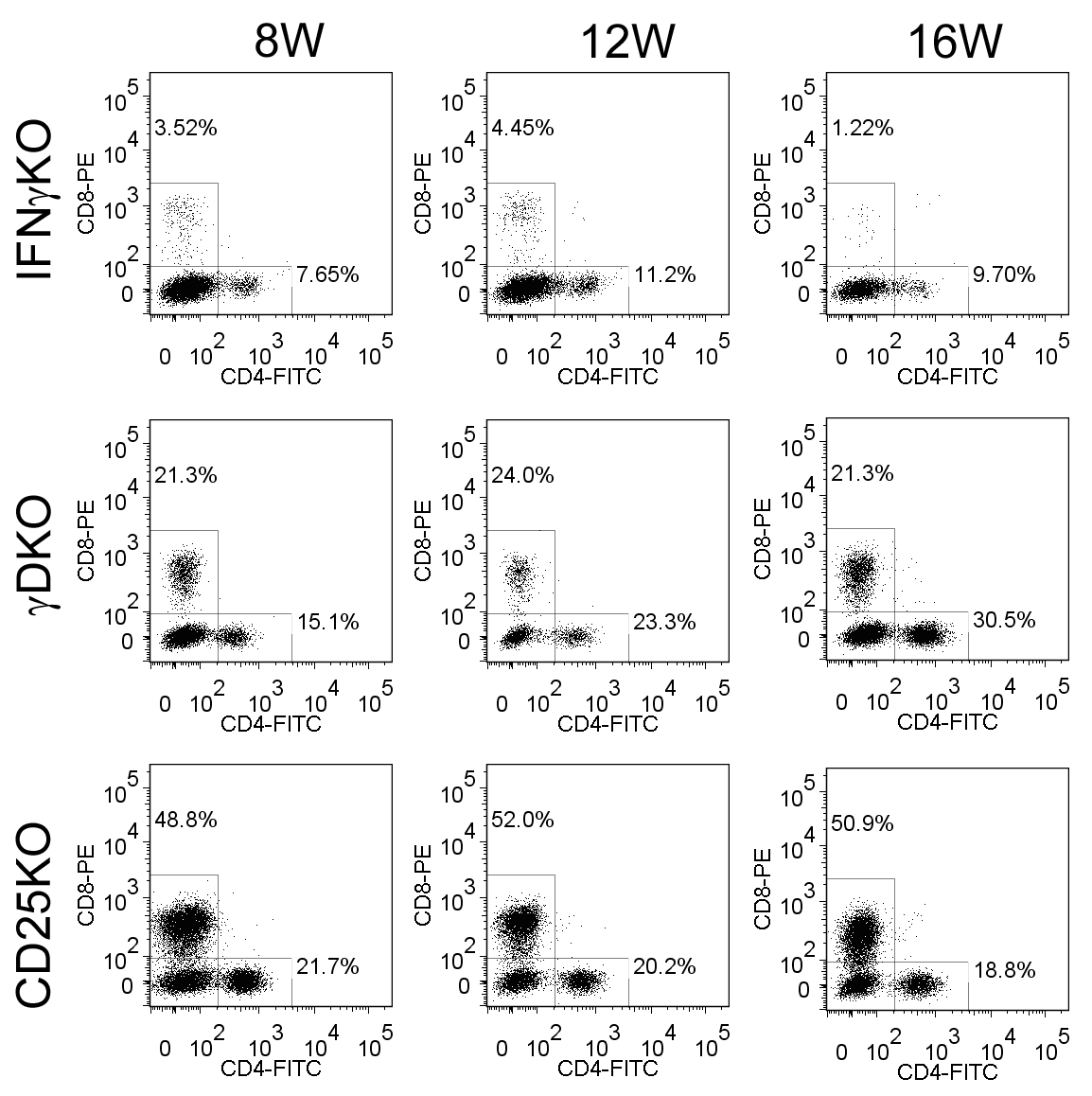

Supplement: Additional file 1 — CD4 and CD8 representative flow cytometry in lacrimal glands (LGs). Representative flow-cytometry plots of freshly isolated cells from LGs of CD25KO, γDKO, and IFN-γKO mice stained with either CD4-FITC-conjugated antibody (x axis) or CD8-PE-conjugated antibody (y axis) at ages 8, 12, and 16 weeks (8W,12W,16W, respectively). Lymphocytes were gated based on characteristic light-scatter properties; single lymphocytes were gated based on forward-scatter height versus forward-scatter area (FSC-A), and dead cells were excluded with propidium iodide staining. [file ar4077-S1.TIFF]
